# Control of Hedgehog Signalling by the Cilia-Regulated Proteasome

**DOI:** 10.3390/jdb4030027

**Published:** 2016-09-03

**Authors:** Christoph Gerhardt, Antonia Wiegering, Tristan Leu, Ulrich Rüther

**Affiliations:** Institute for Animal Developmental and Molecular Biology, Heinrich-Heine University Düsseldorf, 40225 Düsseldorf, Germany; christoph.gerhardt@hhu.de (C.G.); antonia.wiegering@hhu.de (A.W.); tristan.leu@hhu.de (T.L.)

**Keywords:** ciliary proteasome, proteolytic processing, GLI2, GLI3, cilia, signalling, RPGRIP1L, mouse, *Drosophila*, SHH

## Abstract

The Hedgehog signalling pathway is evolutionarily highly conserved and essential for embryonic development of invertebrates and vertebrates. Consequently, impaired Hedgehog signalling results in very severe human diseases, ranging from holoprosencephaly to Pallister-Hall syndrome. Due to this great importance for human health, the focus of numerous research groups is placed on the investigation of the detailed mechanisms underlying Hedgehog signalling. Today, it is known that tiny cell protrusions, known as primary cilia, are necessary to mediate Hedgehog signalling in vertebrates. Although the Hedgehog pathway is one of the best studied signalling pathways, many questions remain. One of these questions is: How do primary cilia control Hedgehog signalling in vertebrates? Recently, it was shown that primary cilia regulate a special kind of proteasome which is essential for proper Hedgehog signalling. This review article will cover this novel cilia-proteasome association in embryonic Hedgehog signalling and discuss the possibilities provided by future investigations on this topic.

## 1. Introduction

In the year 1980, Christiane Nüsslein-Volhard and Eric Frank Wieschaus reported the identification of 15 loci whose mutations affect the development of the fruit fly *Drosophila melanogaster* larvae [1]. In the course of these investigations, the *hedgehog* (*hh*) gene was also discovered. Soon, it was evident that this gene is not only essential for the development of invertebrates, like *Drosophila*, but also for the development of vertebrates [2,3,4,5]. The product of this gene serves as a ligand for an evolutionarily highly-conserved signalling pathway which was termed hedgehog (HH) signalling pathway. Important components of the vertebrate HH signal transduction are the glioblastoma (GLI) proteins—GLI1, GLI2, and GLI3. While GLI1 is a target gene of GLI2-A and GLI3-A, and exclusively acts as a constitutive transcriptional activator, itself [6,7], GLI2 and GLI3 can function as activators or repressors [8], revealing the complex nature of regulating GLI processing. The conversion of the full-length GLI2 and GLI3 proteins into their truncated repressor forms is called proteolytic processing. This processing event is realised by the ubiquitin-proteasome system (UPS).

The UPS is the major protein degradation system in eukaryotes, important for up to 80%–90% of all eukaryotic proteins, revealing its importance [9]. It consists of, in simplified terms, ubiquitin-activating enzymes (E1), ubiquitin-conjugation enzymes (E2), ubiquitin ligases (E3), and the proteasome. Proteins designated to get degraded become phosphorylated and subsequently ubiquitinated. The polyubiquitin conjugation is realised by the cooperative action of E1, E2, and E3. The proteasome is the catalytic component of the ubiquitin-proteasome system consisting of two regulatory 19S subunits and a catalytic 20S subunit. Ubiquitinated proteins are recognised by the 19S regulatory subunits and subsequently degraded by the 20S subunit which contains multiple peptidases [10]. However, not every proteasomal action results in protein degradation. A few proteins get proteolytically processed from their full-length form to a shorter repressor form.

As the UPS is not only important for protein processing, but also for protein degradation, it can influence proteins in different manners. Interestingly, this is also true for HH signalling. For example, GLI2 and GLI3 are proteolytically processed [11], while GLI1 is not processed, but degraded, by the UPS [12,13,14]. Thus, the GLI proteins serve as a very interesting model to analyse the molecular mechanisms underlying the differences between protein degradation and protein processing. While the subcellular localisation where GLI1 gets phosphorylated is unknown, the phosphorylation process of GLI2 and GLI3 is thought to take place at the basal body (BB) of cilia [15]. In the processing event of GLI2 and GLI3, ubiquitination takes place immediately after phosphorylation. However, it is still unknown if the polyubiquitination of GLI2 and GLI3 occurs at the BB. Additionally, the ubiquitin E3 ligase JADE-1, which is known to be involved in the ubiquitination of β-catenin [16], has been shown to be localised at the BB [17]. Furthermore, the key ubiquitin E3 ligase, APC, was found to be located at the BB [18] supporting the hypothesis that both, phosphorylation and polyubiquitination processes take place at the BB.

**Figure 1:**
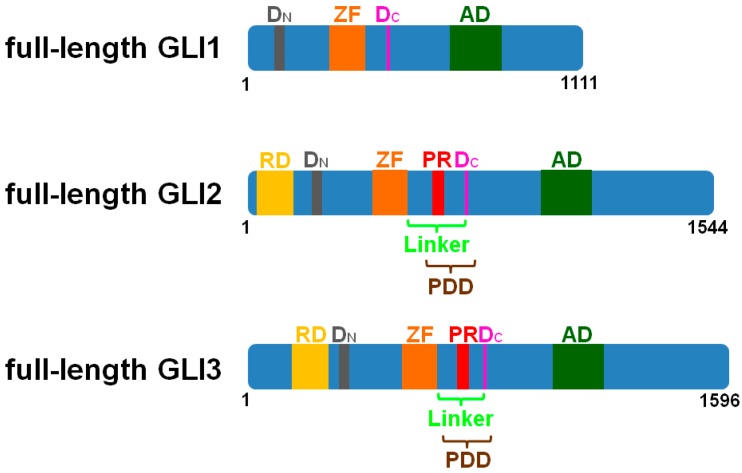
Schematic illustration of domains in murine GLI1, GLI2, and GLI3. GLI1, GLI2, and GLI3 bind to DNA via their zinc-finger domains (ZF). While all three proteins contain an activator domain (AD), only GLI2 and GLI3 display a repressor domain (RD), a processing determinant domain (PDD) and a processing region (PR). The N-terminal degron (D_N_) plays an important role in the degradation of all GLI proteins. The C-terminal degron (D_C_) acts as a degradation signal in GLI1 and as a processing signal in GLI2 and GLI3.

Apart of differences in GLI proteins of being degraded or processed, also differences in efficiency of processing are noticeable. For example, GLI2 is not as efficiently processed as GLI3 [19]. Previous studies, therefore, tried to examine the differences between these proteins in order to understand the reason leading to this variation in processing efficiency. Pan et al. hypothesised that differential GLI2 and GLI3 processing is controlled by the processing determinant domain (PDD) [20] (Figure 1). Accordingly, they could show that replacement of GLI2-PDD by GLI3-PDD led to a more efficient processing of GLI2 [21]. Moreover, Schrader et al. examined the difference between GLI1 and GLI3. They found a three-part signal mechanism which is dependent on a zinc-finger domain, the linker sequence, and the degron, which is essential for GLI3 processing [22] (Figure 1). The first processing signal is the zinc-finger domain of GLI3. However, this domain is not the crucial difference to distinguish proteins from being either processed or degraded, because GLI1 also includes a similar zinc-finger domain. The second processing signal is the linker sequence which is located between the zinc-finger domain and the lysines of the degron sequence. It is proposed that the proteasome binds to the linker area which is assumed to be a proteasome initiation region. Finally the degron is the third processing signal and the starting point of proteasomal processing. The linker sequence in combination with the degron is missing in GLI1 and, hence, seems to be the critical difference leading to processing of GLI3, and not of GLI1 [22]. Consequently, the sequence of the respective protein determines whether it becomes degraded or processed, while the proteasome is not the factor which distinguishes degradation from processing.

In vertebrates, HH signalling is mediated by cytoplasmic protrusions known as primary cilia. Primary cilia have a length of 1–15 μm depending on the cell type and basically consist of three different compartments: the BB, the axoneme and the transition zone (TZ). The BB is a modified centrosome from which the ciliary microtubule-based framework (axoneme) grows out. The axoneme, which is comprised of nine doublet microtubules arranged in a circle, gives stability to the cilium and allows protein transport inside the cilium. The region in between the BB and the axoneme is called TZ. The TZ acts as a selective barrier controlling ciliary import and export of proteins. Ciliary assembly and maintenance relies on the bidirectional microtubule-based intraflagellar transport (IFT) that allows the permanent delivery of axonemal precursors to the assembly site at the ciliary tip [23]. On the basis of their structure, primary cilia function as sensory antennae at the surface of cells by concentrating multiple receptors. These receptors then, in turn, mediate the transduction of many signalling cascades including the HH signalling cascade [24,25].

**Figure 2:**
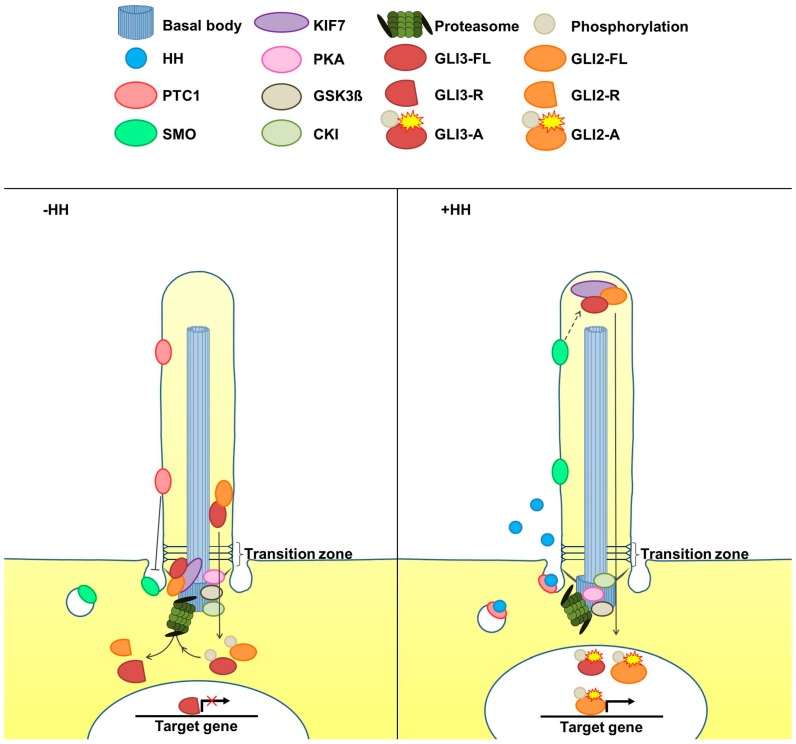
Simplified scheme of the cilia-mediated HH signal transduction pathway in vertebrates. Without HH, PTC1 is located in the ciliary membrane and prevents the ciliary entry of SMO. As a consequence, the full-length proteins of GLI2 and GLI3, which are bound to the ciliary tip organizer KIF7, are phosphorylated by PKA, CK1, and GSK3, and finally proteolytically processed by the cilia-regulated proteasome. KIF7 is essential for this processing event but the mechanism by which KIF7 controls GLI processing remains elusive [36]. The products of GLI2 and GLI3 processing are their repressor forms (GLI2-R and GLI3-R) which enter the nucleus and block HH target gene expression. The repressor form of GLI3 is, thereby, predominant. In the presence of HH, the HH ligand binds to PTC1 and, in turn, the HH/PTC1 complex leaves the cilium allowing ciliary entry of SMO. By a poorly understood mechanism, SMO causes the conversion of the full-length GLI2 and GLI3 proteins (GLI2-FL and GLI3-FL) into GLI2 and GLI3 activator forms (GLI2-A and GLI3-A), which induce HH target gene expression.

In summary, a simplified model of HH signal transduction at the primary cilium is conducted by different proteins (also see Figure 2): The twelve-pass transmembrane receptor PATCHED1 (PTC1) is located in the ciliary membrane of vertebrates. After being bound by its ligand HH, the HH/PTC1 complex leaves the cilium. Subsequently, the seven-pass transmembrane protein SMOOTHENED (SMO) accumulates in the ciliary membrane and converts full-length GLI2 and GLI3 (GLI2-FL and GLI3-FL) into transcriptional activators (GLI2-A and GLI3-A) most likely by modifying them [26,27]. Afterwards, GLI2-A and GLI3-A induce the expression of HH target genes (e.g., GLI1). Without HH, PTC1 remains within the ciliary membrane and SMO is not allowed to enter the cilium. In this case, the full-length GLI2 and GLI3 proteins are proteolytically processed into transcriptional repressors (GLI2-R and GLI3-R) [11], demonstrating that GLI2 and GLI3 processing depends on cilia [28]. In detail, the proteolytic processing event of GLI2 and GLI3 initiates with the phosphorylation of both proteins by protein kinase A (PKA) at the BB [15]. In addition to PKA, casein kinase 1 (CK1) and glycogen synthase kinase 3 (GSK3) are involved in the phosphorylation of GLI2 and GLI3 [19,29]. Although GSK3 was detected at the BB [30], there is no evidence yet whether CK1 and GSK3 phosphorylate GLI2 and GLI3 at the base of cilia. An additional player in regulating GLI processing and activity is the kinesin family member 7 (KIF7) protein which localises along the entire cilium [31,32,33,34,35]. However, the mechanism how KIF7 affects GLI processing is only poorly understood.

## 2. The Cilia-Regulated Proteasome Is Essential for GLI Processing

**Figure 3:**
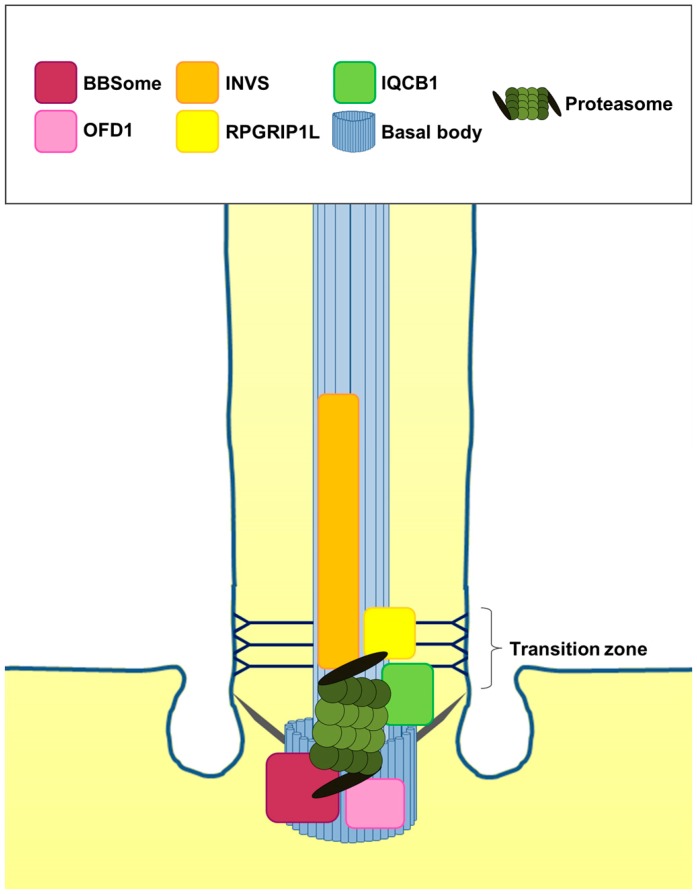
Interactions between ciliary proteins and the cilia-regulated proteasome. INVS is located in the Inversin compartment and in the transition zone and interacts with components of the 19S proteasomal subunit (black lid of the proteasome). The transition zone protein RPGRIP1L interacts with components of the 19S proteasomal subunit. IQCB1 is present at the transition zone and the basal body and interacts with components of the 20S proteasomal subunit. OFD1 localises to the basal body and interacts with components of the 19S proteasomal subunit. Components of the BBSome are located at the basal body and interact with components of the 19S and 20S proteasomal subunit.

In principle, the mechanism of GLI3 processing is well understood, but the subcellular localisation of GLI3 processing is still unknown. As described above, the vertebrate HH signalling machinery tightly depends on primary cilia and all key components of the HH signalling pathway (including GLI2 and GLI3) display a ciliary localisation. In vertebrate cells, proteasomes exist almost ubiquitously within the cytoplasm and the nucleus [37]. Recently, it was shown that proteasomal components localise at the primary cilium. All analysed components of the 19S proteasomal subunit were detected at the BB, while Psma5, a component of the 20S proteasomal subunit, was located along the entire cilium [38]. These localisation studies and the finding that PKA acts at the BB of cilia [15] suggest that the processing of GLI2 and GLI3 takes place at the BB. As the existence of a centrosome-associated proteasome was already shown before [39,40], the question arises whether the cilium is important for the function of the BB-associated proteasome or whether the centrosome-associated proteasome and the BB-associated proteasome are one and the same. In 2003, it was suggested that proteasomes might exert different functions depending on their subcellular localisations and that these differences might be governed by their association and interaction with specific regulatory proteins [41]. Indeed, in silico studies using a systematic network-based approach to work out the “cilia/centrosome complex interactome (CCCI)” showed that the greatest community of the CCCI consists of proteasomal components [42] indicating that the association of ciliary proteins and the proteasome might be of particular significance. Accordingly, the ciliary proteins Bardet-Biedl syndrome (BBS) 1, BBS2, BBS4, BBS6, BBS7, BBS8, inversin (INVS; also known as NPHP2), IQ motif-containing protein B1 (IQCB1; also known as NPHP5), oral-facial-digital syndrome 1 (OFD1), and retinitis pigmentosa gtpase regulator interacting protein 1-like (RPGRIP1L; also known as FTM, NPHP8, or MKS5) interact directly with different proteasomal components [38,43,44,45] (Figure 3). In case of BBS4, Gerdes et al. demonstrated for the first time that a ciliary protein is involved in the proteasomal regulation [46]. It is now known that the loss of BBS4, BBS7, OFD1, or RPGRIP1L leads to a reduced proteasomal activity thereby impairing intercellular signalling pathways [38,43,46,47]. Interestingly, the absence of the proteins BBS4, BBS7, OFD1, or RPGRIP1L result in a severe ciliopathy phenotype in mouse embryos, being associated with multiple defects and increased lethality. For instance, *Bbs4*-negative embryos exhibit defects in brain, eye, kidney, and liver development and become obese [48,49,50,51,52], while *Bbs7*^−/−^ embryos suffer from brain, eye, and sperm defects, as well as from getting obese [53]. *Ofd1*-deficient embryos show defects in brain, limb, lung, heart, and kidney development [54,55], and *Rpgrip1l*-negative murine embryos display brain, eye, limb, lung, heart, kidney, and liver defects [56,57,58]. Analysing effects on lethality, *Bbs7*^−/−^ mice are viable [53], while *Bbs4*^−/−^ mice display a higher perinatal lethality [49] and *Rpgrip1l*^−/−^, as well as *Ofd1*^−/−^, mice are not viable at all [54,55,56].

The proteasome is essential for the development and function of numerous organs and structures of the human body [59,60,61,62,63,64,65,66,67,68,69]. Thus, reduced activity of the cilia-regulated proteasome is a possible cause of ciliopathies. Further evidence highlighting the importance of cilia-regulated proteasomes arises from rescue experiments in vivo. The injection of proteasomal component mRNA or treatment with the proteasome activators mevalonolactone or sulforaphane restored defective convergent extension and somatic definition in zebrafish embryos treated with *bbs4* or *ofd1* morpholinos [43]. However, it would be presumptuous to assume that ciliopathies are exclusively caused by the reduction of proteasomal activity. For example, ciliary length defects can occur independently of the reduced proteasomal activity in *Rpgrip1l*-negative murine embryos [38]. Potentially, the decreased proteasomal activity enhances the severity of a ciliopathy. To test this hypothesis, it would be necessary to quantify proteasomal activity in mouse embryos suffering from milder ciliopathies as for example *Nphp1*-, *Nphp4*-, or *Nek8*-mutant embryos which exclusively display eye or kidney defects [70,71,72]. Moreover, future studies are indispensable to answer the question whether the data about the cilia-regulated proteasome of different model organisms is translatable to humans.

Remarkably, ciliary proteins seem to use different mechanisms with which they regulate proteasomal activity. In the absence of BBS4, BBS7 and OFD1, the reduced proteasomal activity is based on a decreased amount of different proteasomal components revealing that these proteins control the composition of the proteasome [43]. On the contrary, RPGRIP1L deficiency leads to an accumulation of 19S and 20S proteasomal subunit components at the ciliary base [38] indicating that RPGRIP1L regulates proteasomal activity differently. Since RPGRIP1L directs proteasomal activity by the interaction with Psmd2, a component of the 19S proteasomal subunit, it was hypothesised that RPGRIP1L changes the conformation of Psmd2 in order to control proteasomal activity [38]. While analysing the relationship between RPGRIP1L and the proteasome, a novel kind of proteasome was found. Loss of RPGRIP1L provoked a decreased proteasomal activity exclusively at the base of cilia [38]. Consequently, the proteasome located at the base of primary cilia seems to be differently regulated from all proteasomes that are present at other subcellular localisations. Importantly, this kind of proteasome which was termed “ciliary proteasome” is essential for GLI3 processing [38].

## 3. GLI2-R and GLI3-R Are Essential for Proper Embryonic Development in Vertebrates

The relationship between the cilia-regulated proteasome and HH signalling is essential for homeostasis and embryonic development in vertebrates. For example, an alteration of proteasomal activity is thought to be involved in the development of cancer [73,74,75,76,77,78,79,80,81,82,83,84,85,86,87,88,89,90,91,92,93]. In this context, the cilia-regulated proteasome might have an essential role, a topic which was extensively reviewed elsewhere [45]. Here, we will focus on the meaning of HH signalling in vertebrate embryogenesis. Regarding HH signalling, the products of the cilia-regulated proteasome are GLI2-R and GLI3-R. Although it was reported that GLI2 is not processed as efficiently as GLI3 [19], both proteins play decisive roles during vertebrate development in their truncated forms. GLI2-R takes part in the development of the brain and the limbs in mice as well as the neural tube in zebrafish [94,95,96]. Together with GLI3-R, GLI2-R controls anterior limb patterning and digit number [96]. However, the scope of GLI3-R in vertebrate development is much broader than that of GLI2-R. In addition to limb development [97], GLI3-R was shown to be involved in the development of the vertebrate lung, kidneys, ureter, mammary gland, ears, and the CNS. In mice, GLI3-R controls proper lung organogenesis [98], the nephron number [99,100], the functional development of the ureter [101], the induction of mammogenesis [102], and the dorsoventral patterning of the inner ear [103]. As another review article has previously discussed, the cilia-regulated proteasome seems to be involved in most if not all cilia-mediated signalling pathways [45]. Consequently, all defects caused by a decreased activity of the cilia-regulated proteasome are most likely a consequence of numerous disturbed cilia-mediated signalling pathways. However, the outstanding importance of the association between the cilia-regulated proteasome and HH signalling for vertebrate development is highlighted by investigations using the *Gli3*^Δ699^ mouse mutant which is considered to express a constitutively active GLI3-R protein. The *Gli3*^Δ699^ allele was generated by an insertion of a selection marker cassette into the *Gli3* locus [104]. This mutant allele terminates just C-terminally of the zinc-finger domain (amino acid position 699). *Gli3*^Δ699*/+*^ mice are viable and fertile with exhibiting polydactyly of the forelimbs at very low frequency. However, *Gli3*^Δ699*/*Δ699^ mice died within 12–18 h after birth. Mouse embryos homozygous for *Gli3*^Δ699^ suffer from skeletal and limb defects, imperforate anus, visceral abnormalities, loss of the adrenal gland, and kidney defects. Remarkably, the introduction of GLI3^Δ699^ restores several aspects of brain development, like telencephalic patterning, olfactory bulb morphogenesis, and the agenesis of the corpus callosum in *Rpgrip1l*-negative mouse embryos [105,106].

Until now, the existence of the cilia-regulated proteasome was reported in the model organisms mouse and zebrafish [38,43], suggesting that this kind of proteasome is present in all vertebrates. However, to consider the impairment of the cilia-controlled proteasome as a candidate factor for human diseases (e.g., ciliopathies or cancer), it is inevitable to perform future experiments demonstrating the existence of a cilia-regulated proteasome in humans. To analyse the link between the cilia-governed proteasome and HH signalling, these future studies should also address the question whether human GLI2 and GLI3 are processed by this kind of proteasome.

## 4. Is the Role of the Cilia-Regulated Proteasome Evolutionarily Conserved?

The cilia-regulated proteasome is essential for HH signalling and for embryonic development in mice [38,43]. Until 2014, the general view was that HH signalling was extremely important for embryonic development of the invertebrate *Drosophila*, but operating in a cilia-independent manner [107]. It was thought that HH signalling in *Drosophila* mainly occurs over the entire plasma membrane of its cells. Thus, PTC localises at any place of the plasma membrane and inhibits the presence of SMO in the membrane. The binding of HH to PTC allows SMO to enter the plasma membrane after membrane exit of the HH/PTC complex. As a consequence, cubitus interruptus (CI), the *Drosophila* homolog of the GLI proteins, is converted into CI-A in the cytoplasm. In the absence of HH, CI is processed to CI-R by proteasomes in the cytoplasm [108,109,110,111,112,113]. To our knowledge, a specific subcytoplasmic localisation of these proteasomes has never been reported. After the conversion of CI, CI-A or CI-R translocates into the nucleus to regulate HH target gene expression [113,114,115]. In contrast to vertebrates, *Drosophila* seems to form cilia exclusively on sensory neurons and spermatozoa [116,117]. In 2014, Kuzhandaivel et al. reported the transduction of HH signals by the immotile cilia of olfactory sensory neurons (OSNs) in *Drosophila* [118]. PTC, SMO, and COS2 were demonstrated to localise along the whole cilium of OSNs and it was shown that the ciliary presence of SMO is important for proper HH signalling. Although the general view is that HH signalling cannot function without CI in *Drosophila* [119], CI was not detectable in OSN cilia [118]. However, ciliary localisation of SMO induces the expression of HH target genes in OSNs. Since CI gets proteolytically processed by the proteasome [108,109,110,111,112,113], the question arises whether the proteasome which processes CI is regulated by ciliary proteins in *Drosophila* OSNs. Until now, it is even unknown whether proteasomes are present at the base of OSN cilia. A detection of proteasomal components at the base of OSN cilia would make the existence of an evolutionarily conserved cilia-regulated proteasome likely. If future investigations demonstrated that proteasomal components are absent from the ciliary base of OSNs, it would be interesting to analyse where the processing of CI takes place in OSNs. Furthermore, if proteasomal components are missing at the base of OSN cilia, the fact that CI was not detected in OSN cilia [118] raises the question whether HH signal transduction in OSN cilia is able to function without CI. Since this finding would be rather surprising, and highly interesting, it should be a topic of future studies. However, previous studies could show an association between HH signalling and cilia in planarians [120], but the localisation of HH pathway components at cilia or even the mediation of HH signalling by cilia was never shown for other invertebrates than *Drosophila*. The fact that RPGRIP1L, the ciliary protein being known to exclusively control the cilia-localised proteasome in mice [38], is missing in *Drosophila* [56] gives two possibilities: the first possibility is that the cilia-regulated proteasome does not exist in *Drosophila* and, consequently, RPGRIP1L is not needed in *Drosophila*. The second possibility is that other proteins than RPGRIP1L control the activity of the cilia-governed proteasome in *Drosophila*. If future studies in vertebrates lead to the identification of new proteins that control the cilia-regulated proteasome, the presence of these proteins should be analysed in *Drosophila*. Furthermore, it would be of great interest to understand how these ciliary proteins control the ciliary proteasome. These findings might reveal a conserved regulatory mechanism in evolution, but if *Drosophila* lacks the cilia-controlled proteasome, another hypothesis is conceivable: Since RPGRIP1L is conserved from cnidarians to humans, but is absent in arthropods and nematodes [56], *Drosophila* could be an exception regarding the presence of the cilia-regulated proteasome. Remarkably, PSMA5, a component of the 20S proteasomal subunit, was detected along the whole cilium in mice [38]. Within flagella of the green alga *Chlamydomonas reinhardtii*, the components of the ubiquitin conjugation system, but no proteasomal components, have been found [121] suggesting that the cilia-localised proteasome might have developed later in evolution.

## 5. Is the Cilia-Regulated Proteasome Involved in the Regulation of PTC and/or SMO Action?

Even at the level of PTC and SMO, proteasomes exert essential functions in HH signalling. In vitro studies in vertebrate systems using primary cells, as well as cell lines from different vertebrate species, demonstrated that, in the presence of HH, PTC1 was ubiquitinated at K1413 in its C-terminal domain by the E3 ligases ITCH and WWP2 and finally degraded by the proteasome [122]. In the absence of HH, SMO is not allowed to enter the cell membrane and to localise at the cell surface. Several examinations in *Drosophila* revealed that SMO gets ubiquitinated, internalised, and degraded by lysosome- and proteasome-dependent mechanisms in lack of HH [123,124,125,126]. Interestingly, SMO is able to form a complex with an E3 ligase called SMURF which directly controls proteasomal degradation of HH-unbound PTC [127].

Consequently, positive, as well as negative, regulation of HH signalling depends on proteasomal intervention. Due to the close relation of vertebrate HH signalling to cilia, the question arises whether the cilia-regulated proteasome or another kind of proteasome realises the degradation of PTC and/or SMO. Considering that loss of RPGRIP1L reduces exclusively the activity of the BB-localised proteasome, SMO would be expected to accumulate at cilia in the absence of RPGRIP1L. However, the localisation of SMO is unaltered upon RPGRIP1L deficiency [38] suggesting that the cilia-regulated proteasome might not be responsible for the degradation of SMO. To definitely evaluate the contribution of the cilia-regulated proteasome, it is imperative that further investigations take place.

## 6. Conclusions

The HH signalling transduction cascade is involved in the development of almost every vertebrate organ and structure [128]. Consequently, impaired HH signalling results in severe human diseases [24,129,130,131]. In this context, the precise coordination between positive and negative regulation of HH signalling is of eminent importance. Since it is yet unknown whether the cilia-regulated proteasome is involved in the regulation of PTC and SMO, this review focuses on the negative regulation mechanisms via proteolytic processing of the GLI2 and GLI3 transcription factors by the cilia-regulated proteasome. Two major questions have to be addressed on this topic:

(1) Is the proteolytic processing of the GLI2 and GLI3 proteins by the cilia-regulated proteasome evolutionarily conserved?

The HH transduction pathway is evolutionarily highly conserved. In vertebrates, HH signalling is mainly transduced by primary cilia. Within this cilia-mediated HH signalling, the cilia-regulated proteasome plays a decisive role. Previously, it was shown that cilia of sensory neurons are able to mediate HH signalling [118]. Thus, *Drosophila* is a well-suited model organism to examine if the cilia-regulated proteasome also exists in invertebrates and, if this is the case, to analyse whether the proteolytic processing of CI is facilitated by this kind of proteasome. Since several experiments are easier to perform in *Drosophila* than in mice, the presence of the cilia-controlled proteasome in *Drosophila* could give the opportunity to gain more insight into the mechanisms underlying the regulation of this proteasome by ciliary proteins.

(2) Does the cilia-regulated proteasome play a role in the control of HH signalling during human embryonic development?

As early as 1995, Denys Wheatley proposed primary cilia to be necessary for human homeostasis [132]. Today, the outstanding importance of primary cilia for human development is indisputable, but it has to be shown whether the cilia-regulated proteasome participates in human embryonic development. If this is true, it should be analysed whether this participation is involved in the control of HH signalling (GLI3 processing) and to what extent the cilia-governed proteasome affects embryonic development in humans. The precondition for these studies is the existence of the cilia-controlled proteasome in humans. Previously, interactions between proteasomal components and ciliary proteins have been shown in murine as well as in human cells [38,43,44] indicating that the cilia-regulated proteasome might exist in humans. If future studies reveal that the cilia-regulated proteasome is essential for human embryonic development, novel therapies against severe human ciliopathies are possible. In this context, proteasome activators were successfully used for treatment of ciliopathic zebrafishs and cells isolated from ciliopathic mice [38,43]. It is plausible that a prenatal application of proteasome activators to pregnant women could be a curative treatment of ciliopathies in the future.
